# The mitochondrial genome of *Faughnia haani* (Stomatopoda): novel organization of the control region and phylogenetic position of the superfamily Parasquilloidea

**DOI:** 10.1186/s12864-021-08034-x

**Published:** 2021-10-02

**Authors:** Hee-seung Hwang, Jongwoo Jung, Juan Antonio Baeza

**Affiliations:** 1 Research Institute of EcoScience, Ewha Womans University, Seoul, 03760 Republic of Korea; 2 Division of EcoCreative, Ewha Womans University, Seoul, 03760 Republic of Korea; 3 Department of Science Education, Ewha Womans University, Seoul, 03760 Republic of Korea; 4 Department of Biological Sciences, 132 Long Hall, Clemson University, Clemson, SC 29634 USA; 5 Smithsonian Marine Station at Fort Pierce, 701 Seaway Drive, Fort Pierce, Florida, 34949 USA; 6 Departamento de Biología Marina, Facultad de Ciencias del Mar, Universidad Católica del Norte, Larrondo, 1281 Coquimbo, Chile

**Keywords:** Control region organization, Raptorial claw, Mitochondrial genome, Parasquilloidea, Stomatopoda, Vision

## Abstract

**Background:**

Stomatopod crustaceans are aggressive marine predators featuring complex compound eyes and powerful raptorial appendages used for “smashing” or “spearing” prey and/or competitors. Among them, parasquilloids (superfamily Parasquilloidea) possess eyes with 2-3 midband rows of hexagonal ommatidia and spearing appendages. Here, we assembled and analyzed the complete mitochondrial genome of the parasquilloid *Faughnia haani* and explored family- and superfamily-level phylogenetic relationships within the Stomatopoda based on mitochondrial protein coding genes (PCGs).

**Results:**

The mitochondrial genome of *F. haani* is 16,089 bp in length and encodes 13 protein coding genes (PCGs), 22 transfer RNA genes, 2 ribosomal RNA genes, and a control region that is relatively well organized, containing 2 GA-blocks, 4 poly-T stretches, various [TA(A)]n-blocks, and 2 hairpin structures. This organized control region is likely a synapomorphic characteristic in the Stomatopoda. Comparison of the control region among superfamilies shows that parasquilloid species are more similar to gonodactyloids than to squilloids and lysiosquilloids given the presence of various  poly-T stretches between the hairpin structures and [TA(A)]n-blocks. Synteny is identical to that reported for other stomatopods and corresponds to the Pancrustacea ground pattern. A maximum-likelihood phylogenetic tree based on PCGs revealed that Parasquilloidea is sister to Lysiosquilloidea and Gonodactyloidea and not to Squilloidea, contradicting previous phylogenetic studies.

**Conclusions:**

The novel phylogenetic position of Parasquilloidea revealed by our study indicates that ‘spearing’ raptorial appendages are plesiomorphic and that the ‘smashing’ type is either derived (as reported in previous studies) or apomorphic. Our results raise the possibility that the spearing raptorial claw may have independently evolved twice. The superfamily Parasquilloidea exhibits a closer relationship with other stomatopod superfamilies with a different raptorial claw type and with dissimilar numbers of midband rows of hexagonal ommatidia. Additional studies focusing on the assembly of mitochondrial genomes from species belonging to different genera, families, and superfamilies within the order Stomatopoda are warranted to reach a robust conclusion regarding the evolutionary history of this iconic clade based on mitochondrial PCGs.

**Supplementary Information:**

The online version contains supplementary material available at 10.1186/s12864-021-08034-x.

## Background

**Fig. 1:**
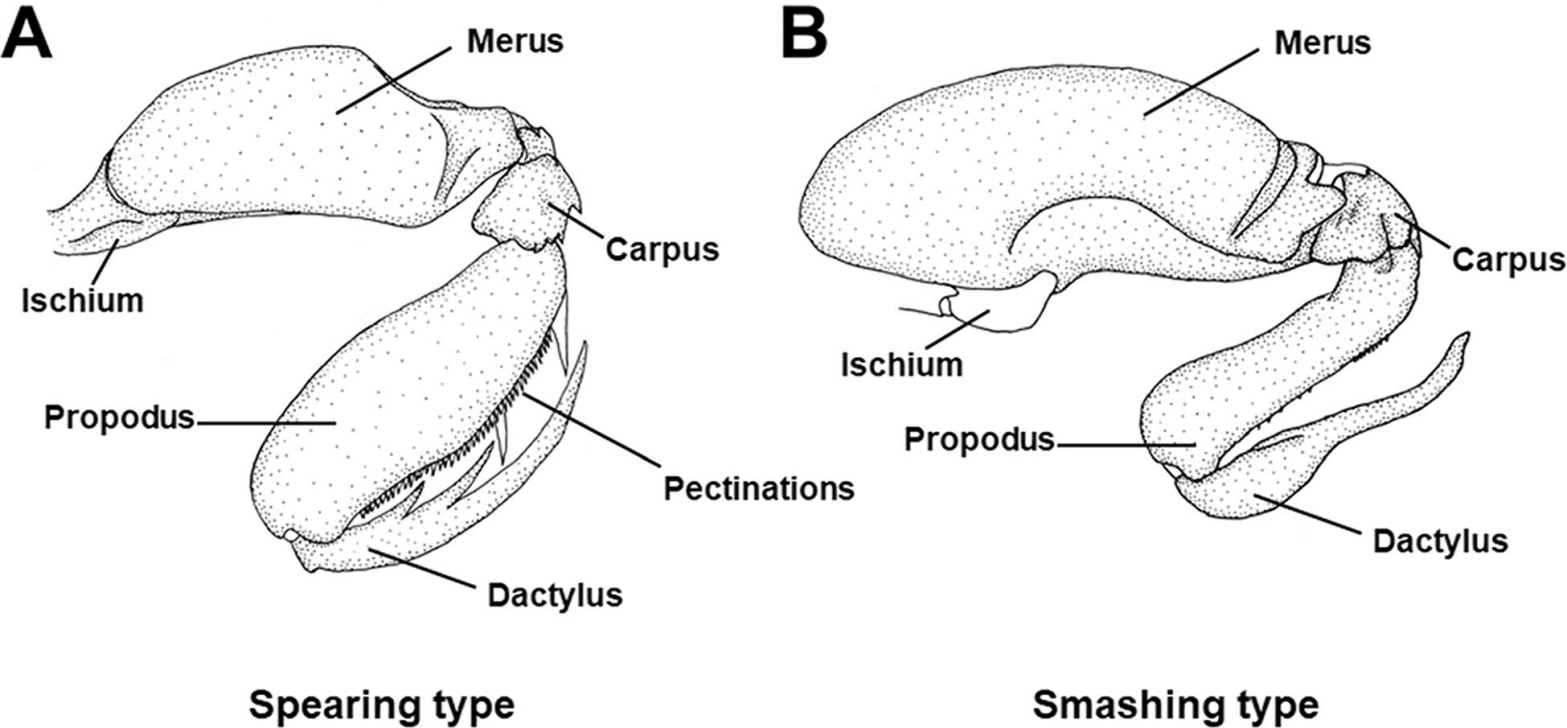
Morphology of the two types of raptorial appendages. A, Spear species, *Faughnia haani*. B, Smasher species, *Gonodactylaceus falcatus*

Mantis shrimps (order Stomatopoda) are aggressive malacostracan crustaceans, which are mainly found in tropical and subtropical regions [1, 2]. More than 480 species in 7 superfamilies and 17 families have been discovered [1, 3, 4]. Stomatopods play important roles in numerous marine ecosystems because the considerable biomass they attain and their position in the trophic web that most likely affect the biomass of other benthic organisms; they are recognized as both important prey and predators [5]. In all stomatopods, the second maxillipeds are modified as powerful raptorial appendages that can be used either for “spearing” or “smashing” prey and/or competitors [6]. Species bearing the two types of appendages differ in terms of morphology, habitat, behaviour, and preferred prey type [7, 8] (Figs. 1A-B). In spearer stomatopods, the dactylus of the raptorial claws possesses teeth and the propodus is generally spined or has pectinations (Fig. 1A). Spearer stomatopods usually thrive at relatively deep depths, are nocturnal, live in burrows in soft sediment, and prefer soft-bodied prey. In turn, the dactylus of smasher stomatopods is devoid of teeth, and the propodus is usually smooth with an inflated proximal end (Fig. 1B) which functions as a hammer. Smasher stomatopods live in shallow waters, are diurnal, live in crevices in hard substrate, and prefer hard-bodied prey [7–10]. The raptorial claw of smasher stomatopods moves at a speed of 14–23 ms^− 1^ and generates extremely high forces under acceleration conditions [11], while that of spearer stomatopods strikes with relatively slow speeds of 2–7 m s^− 1^; however, the elongated morphology of the spearer raptorial appendage compared to that of the smasher appendage implies that spearer mantis shrimps can reach longer distances to attack prey than smasher stomatopods [12–14]. Smasher stomatopods, which are relatively faster and more powerful than spearers, are more likely to capture and to feed upon a wide variety of preys [14, 15]. It has been suggested that the spearing raptorial claw evolved from an ancestral species  bearing a smashing raptorial appendage [15].

Stomatopods also possess apposition compound eyes that contain more photoreceptor types than any other known animal [16]. A recent phylogenetic study based on several short nuclear and mitochondrial gene markers and ancestral state reconstructions indicated that the most recent common ancestor of extant stomatopods had eyes with six midband rows of hexagonal ommatidia [17, 18]. Most extant stomatopod species bear six rows; however, the midband is absent in some groups or is composed of two or three rows of ommatidia in other clades. Bathysquilliods have no midband. Squilloids, *Faughnia* Serène, 1962, and *Parasquilla* Manning, 1961 belonging to the family Parasquillidae; *Coronidopsis* Hansen, 1926 and *Manningia* Serène, 1962 belonging to Eurysquillidae; and some Lysiosquillids have two midband rows. One Parasquillidae species, namely *Pseudosquillopsis* Serène, 1962, bears three midband rows [19]. The aforementioned disparity in eye complexity indicates that reduction in visual elements proceeded independently in different stomatopod lineages. Most likely, eye complexity is influenced by the visual environment, in turn, characterized by dissimilar levels of light (depth) and water turbidity [2].

Within the Stomatopoda, the superfamily Parasquilloidea includes a single family, Parasquillidae, comprising 13 species in three different genera (*Parasquilla*, *Faughnia*, and *Pseudosquillopsis*) [1, 3]. The number of midband rows of hexagonal ommatidia is genus-specific. *Faughnia* and *Parasquilla* have two midbands, while *Pseudosquillopsis* has three midbands [18–20]. *Faughnia* and *Parasquilla* inhabit relatively deep environments (range: 45–200 m), while representatives of the genus *Pseudosquillopsis* inhabit shallow coastal areas [1, 21]. Thus, the reduction in the number of midband rows of hexagonal ommatidia seems to be an adaptive trait in this clade, with more midband rows in shallow versus deep water species [2].

Regarding the phylogenetic position of the currently recognized superfamily Parasquilloidea, the family Parasquillidae was originally placed in the superfamily Gonodactyloidea, because of shared morphological attributes including a subcylindrical body shape, the number of telson denticles, and the maxilliped and post larval morphology, all of which are considered plesiomorphic traits in the Stomatopoda [3, 19]. However, recent phylogenetic studies based on morphological traits [1, 19] and a limited number of molecular characters (i.e., fragments of the mitochondrial genes *cox1*, 12S, and 16S, and/or the nuclear 18S and 28S ribosomal RNA genes) [17, 18] showed that Parasquillidae was a separate superfamily, having a close relationship to the superfamily Squilloidea. Considering that both parasquilloids and squilloids have a spearing-type raptorial appendage and similar midbands rows, these characteristics may be homologous [1, 19].

In this study, we assembled and examined the complete mitochondrial genome of a parasquilloid species, *Faughnia haani.* Specifically, we analyzed the nucleotide composition and codon usage profiles of the protein-coding genes (PCGs). We also described each tRNA gene’s secondary structure and examined the putative D-loop/control region in detail. Additionally, we examined the phylogenetic position of *F. haani* among other stomatopods, based on an amino acid alignment of all mitochondrial PCGs. The analysis informs and help understanding the evolution of raptorial appendages and vision in the Stomatopoda.

## Results & discussion

### Mitogenomic architecture and characteristics

**Fig. 2:**
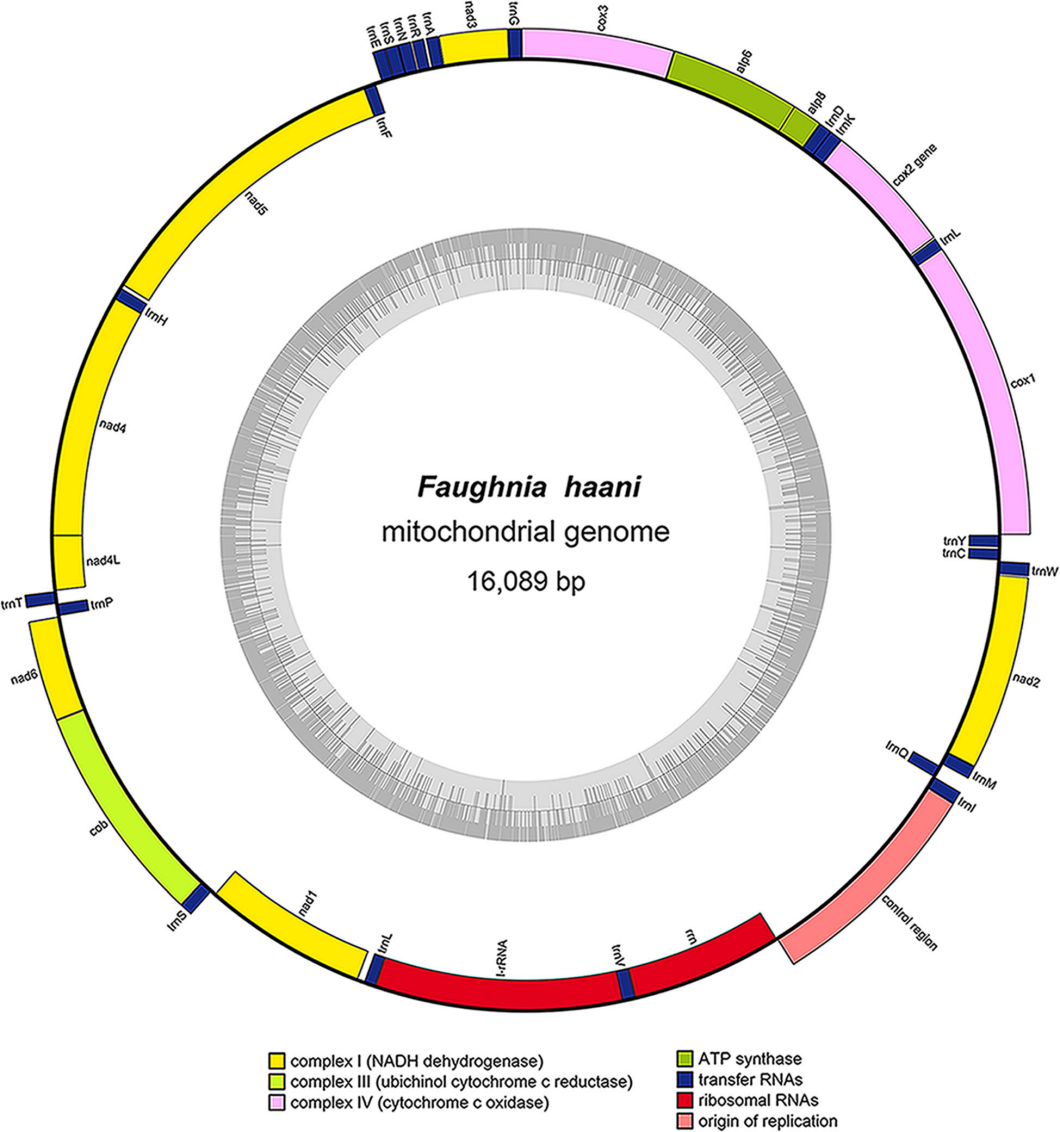
Circular genome map of *Faughnia haani* mitochondrial DNA. The annotated map depicts 13 protein-coding genes (PCGs), two ribosomal RNA genes (rrnS: 12 S ribosomal RNA and rrnL: 16S ribosomal RNA), 22 transfer RNA (tRNA) genes, and the putative control region. The inner circle depicts GC content along the genome. The putative D-Loop/control region is not annotated. Genome assembly and initial nnotation were done with NOVOPlasty and MITOS, and visualized with OrganellarGenomeDRAW

The complete mitochondrial genome of *F. haani* is 16,089 bp in length and encodes 13 protein coding genes, 22 transfer RNAs, and two ribosomal RNAs, plus a putative control region (Fig. 2, Table. 1). The overall nucleotide composition of the mitochondrial genome’s heavy DNA strand was as follows: A = 31.1%, G = 14.6%, C = 19.4%, and T = 35.0%, with a 34.0% G + C content. The mitochondrial genome of *F. haani* contained an AT-skew with an overall base composition on the light strand as follows: A = 40.6%, T = 27.9%, C = 19.8%, and G = 11.7%. Such overall A + T content is within the range described for other arthropods, including crustaceans and other stomatopods [22]. *Squilloides leptosquilla*, belonging to the Squillioidea, is known to exhibit the greatest A + T content among stomatopods (71.2%) [23], while *Squilla empusa* (NC007444) belonging to the Squillioidea and *Lysiosquillina maculata* (NC007443) belonging to the Lysiosquilloidea, exhibit the lowest A + T content (63.90%) [22, 24] (Additional file 1).

**Table 1:** Arrangement and annotation of the mitochondrial genome of *Faughnia haani*

Name	Type	Start	Stop	Strand	Length (bp)	Start	Stop	Inter-genic space	Overlap
*C cox1*	Coding	1	1539	+	1539	ACG	TAA		5
trnL2(tta)	tRNA	1535	1601	+	67			3	
*C cox2*	Coding	1604	2311	+	708	ATG	TAA		19
trnK (aaa)	tRNA	2293	2360	+	68				1
trnD (gac)	tRNA	2360	2426	+	67			8	
*atp8*	Coding	2435	2584	+	150	ATA	TAA		4
*atp6*	Coding	2578	3255	+	678	ATG	TAA		1
*cox3*	Coding	3255	4043	+	789	ATG	TAA	0	
trnG (gga)	tRNA	4044	4112	+	69				15
*nad3*	Coding	4127	4468	+	342	ATA	TAA		0
trnA (gca)	tRNA	4469	4533	+	65			9	
trnR (cga)	tRNA	4543	4607	+	65			2	
trnN (aac)	tRNA	4610	4679	+	70				1
trnS1(agt)	tRNA	4679	4747	+	69				
trnE (gaa)	tRNA	4748	4814	+	67			32	
trnF (ttc)	tRNA	4847	4914	–	68				1
*nad5*	Coding	4910	6573	–	1659	ATG	TAA	71	
trnH (cac)	tRNA	6645	6711	–	67				1
*nad4*	Coding	6727	7923	–	1218	ATG	TAA	7	
*nad4L*	Coding	8306	8046	–	261	ATA	TAA	2	
trnT (aca)	tRNA	8347	8414	+	68				
trnP (cca)	tRNA	8415	8482	–	68			17	
*nad6*	Coding	8500	9024	+	525	ATA	TAA	22	
*cob*	Coding	9047	10,141	+	1095	ATA	TAA		1
trnS2(tca)	tRNA	10,141	10,210	+	70			24	
*nad1*	Coding	11,164	10,235	–	930	ATA	TAA	9	
trnL1(cta)	tRNA	11,174	11,238	–	65				0
rrnL (16S)	rRNA	11,189	12,593	–	1405	GAA	TTA		4
trnV (gta)	tRNA	12,592	12,663	–	72				0
rrnS (12S)	rRNA	12,662	13,496	–	835	TAA	GCA		
D-loop		13,497	14,658	+	1162				
trnI (atc)	tRNA	14,659	14,726	+	68				3
trnQ (caa)	tRNA	14,724	14,792	–	69			12	
trnM (atg)	tRNA	14,805	14,873	+	69			54	
*nad2*	Coding	14,928	15,875	+	948	ATG	TAA	4	
trnW (tga)	tRNA	15,880	15,948	+	69			4	
trnC (tgc)	tRNA	15,953	16,015	–	63			7	
trnY (tac)	tRNA	16,023	16,089	–	67				

The mitochondrial genome of *F. haani* was compact with only a few intergenic spaces and overlaps among gene junctions (Fig. 2, Table. 1). Most PCGs and tRNA genes were encoded on the heavy strand, while only four PCGs (in order from 5′ to 3′: *nad1*, *nad4*, *nad4l*, and *nad5*), two ribosomal RNA genes, and 8 tRNA genes (*trnC*, *trnH*, *trnF*, *trnP*, *trnL*, *trnQ*, *trnV*, and *trnY*) were encoded in the light strand (Fig. 2, Table. 1). A single, long intergenic space which was 1162 bp in length was assumed to be the D-loop/control region (Fig. 2, Table. 1). To date, the mitochondrial genome of twelve species of stomatopod have been assembled: five species belonging to the superfamily Squilloidea Latreille, 1802 [*Squilla empusa* Say, 1818; *Squilla mantis* Say, 1818; *Squilloides leptosquilla* Manning, 1968; *Oratosquilla oratoria* (De Haan, 1844); *Harpiosquilla harpax* (de Haan, 1844); *Lophosquilla costata* (de Haan, 1844)]; two species belonging to the Lysiosquilloidea Giesbrecht, 1910 [*Lysiosquillina maculata* (Fabricius, 1793)]; four belonging to the Gonodactyloidea Giesbrecht, 1910 [*Chorisquilla orientalis* Hwang, Ahyong and Kim 2018, *Taku spinosocarinatus* (Fukuda, 1909), *Gonodactylus chiragra* (Fabricius, 1781)*, Gonodactylaceus randalli* (Manning, 1978)]; and the present species belonging to the superfamily Parasquilloidea Manning, 1995 [*Faughnia haani* (Holthuis, 1959)]. Gene composition and arrangement (synteny) in the mitochondrial genome of *F. haani* is identical to that previously reported for the other eleven stomatopods, and this synteny is consistent with the Pancrustacea (Crustacea + Hexapoda) ground pattern [25].

### Gene features

In the mitochondrial genome of *F. haani*, 12 out of the 13 PCGs exhibited conventional invertebrate and Pancrustacea mitochondrial start codons (ATA and ATG) (Table 1). *cox1* featured an alternative putative start codon (ACG) that has been observed in other malacostracan (Malacostraca) mitochondrial genomes [26, 27] and other stomatopod mitochondrial genomes [22, 28–31]. All thirteen PCGs were observed to end with a complete and conventional stop codon (TAA or TAG), in agreement to that observed in other stomatopod mitochondrial genomes [22, 23, 28–31] (Table 1).

The most frequently used codons in PCGs included UUA (Leu, *N* = 268), UUU (Phe, *N* = 221), and AUU (Ile, *N* = 218). The least frequently used codons (excluding termination codons) were CCG (Pro, *N* = 9), UGC (Cys, *N* = 8), CGC (Arg, *N =* 8), and UCG (Ser, *N =* 8) (Additional file 2). Codon usage in *F. haani* is similar to that observed in *Oratosquilla oratoria* (superfam. Squillioidea) as well as other crustaceans [28, 32, 33].

### Transfer RNA and ribosomal RNA

**Fig. 3:**
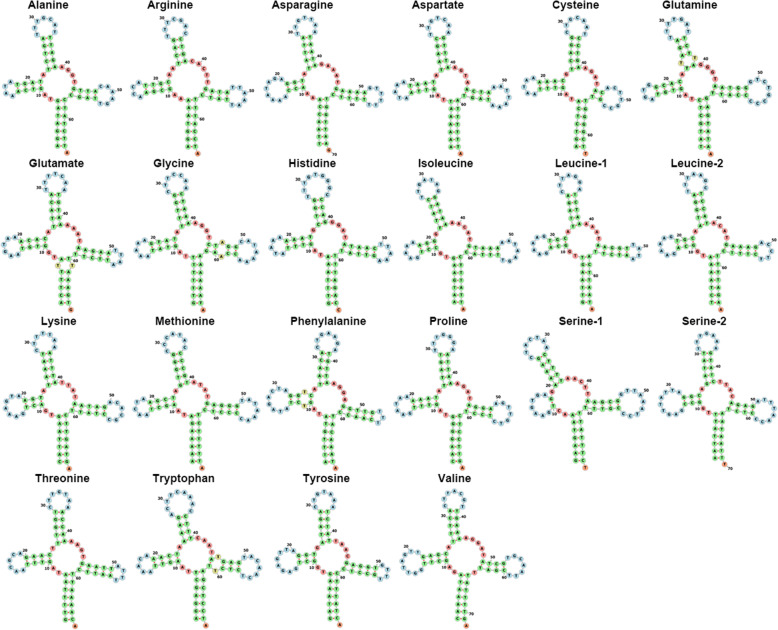
Secondary structure of tRNAs in the mitochondrial genome of *Faughnia haani* visualized in the Forna web server (http://rna.tbi.univie.ac.at/forna) [37]

The mitochondrial genome of *F. haani* encoded tRNA genes that ranged from 63 bp (tRNA-Cys) to 72 bp (tRNA-Val) in length. All tRNA genes, except tRNA-Ser1, exhibited a standard ‘cloverleaf’ secondary structure as predicted by MIFTI (Fig. 3). The dihydrouridine arm of the tRNA-Ser1 gene was missing the stem but not the loop. Truncated tRNAs have often been observed in other stomatopod species (*Squilla mantis* [22], *O. oratoria* [28]) and other metazoan species [34]. Truncated tRNAs have been reported in other decapod crustaceans [32, 33, 35]. The function of these truncated tRNAs seems to be complemented by aminoacylation and EF-Tu (elongation factor thermo unstable) binding [36].

The *LrRNA* and *SrRNA* gene lengths were 1357 and 833 bp, respectively. These genes were located close to each other between tRNA-L and the putative D-loop/CR, but were separated by tRNA-V (Fig. 2, Table. 1). As observed in other crustaceans, including stomatopod species, the two genes exhibited a considerable AT-skew. The overall base composition of the *rrnL* gene was A = 38.9%, T = 34.4%, C = 16.4%, and G = 10.3%. Meanwhile, that of the *rrnS* gene was A = 34.3%, T = 35.5%, C = 19.0%, and G = 11.2%.

### Control region organization

The intergenic region, which was 1162 bp long and assumed to be the D-loop/CR, was located between the 12S ribosomal RNA and tRNA-I (Fig. 2, Table 1). The region was AT-rich with an overall base composition of A = 41.0%, T = 33.0%, C = 17.3%, and G = 8.7%.

**Fig. 4:**
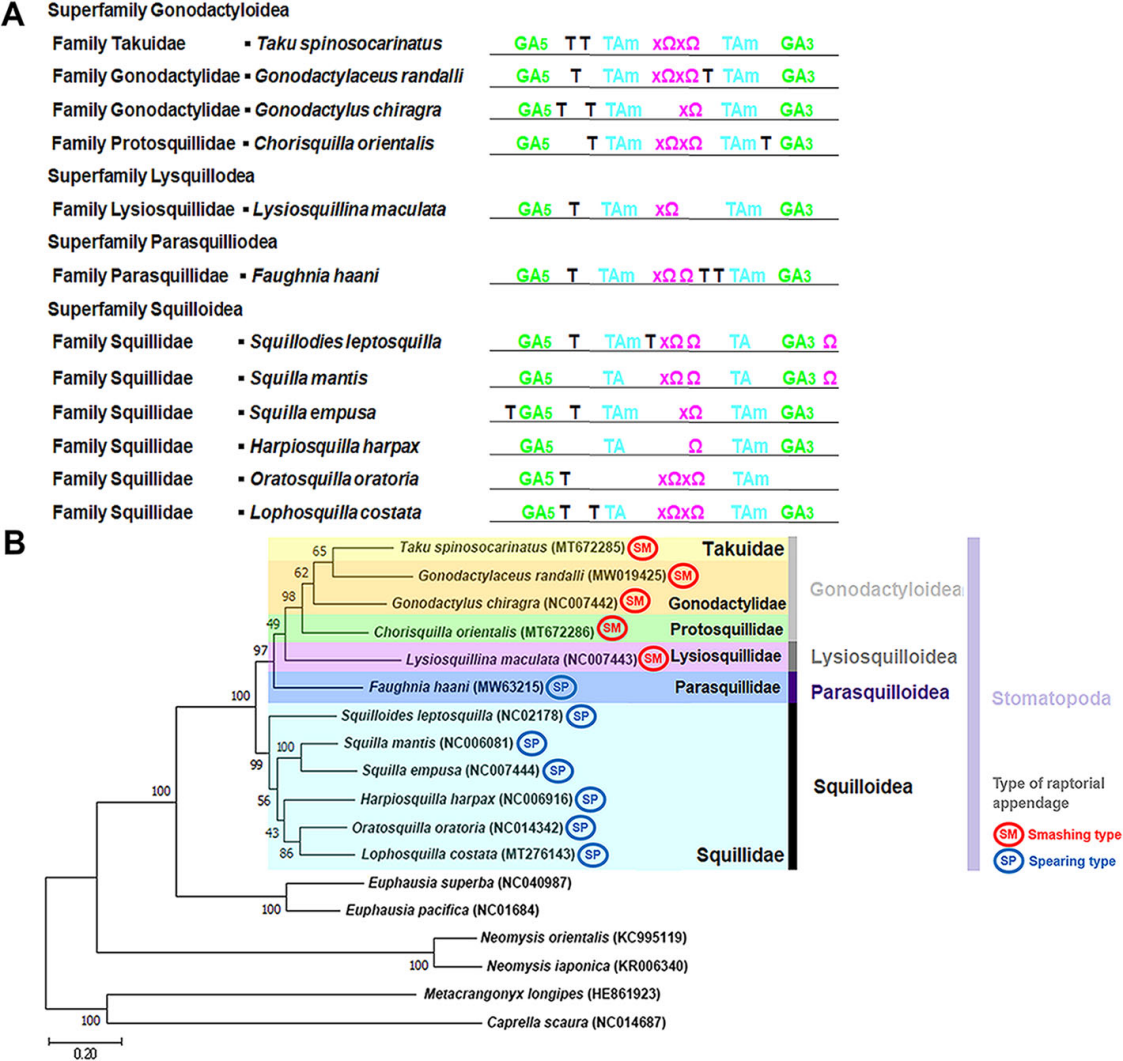
A, Schematic drawing of conserved elements in the control region of twelve stomatopods. B, Maximum-likelihood tree based on the amino acid alignment of 13 PCGs in the stomatopod mitochondrial genome. A, T = poly T stretch, GA5 = GA-5′-block, GA3 = GA-5′-block, TAm means that the [TA(A)]n-block is present multiple times. Ω = potential hairpin structure. An X in front of the Ω indicates that the conserved motif “TTAT” is present. B, Maximum-likelihood phylogenetic tree based on the amino acid alignment of 13 protein coding genes in the mitochondrial genome of stomatopods. Phylogenetic analyses included a total of 11 genera from 6 different families and 4 superfamilies in the order Stomatopoda: *Squilla empusa* (NC007444), *Squilla mantis* (NC006081), *Squilloides leptosquilla* (NC027178), *Lophosquilla costata* (MT276143), *Oratosquilla oratoria* (NC014342), *Harpiosquilla harpax* (NC006916), *Chorisquilla orientalis* (MT672286), *Taku spinosocarinatus* (MT672285), *Lysiosquillina maculata* (NC007443), *Gonodactylus chiragra* (NC007442)*, Gonodactylaceus randalli* (MW019425), and *Faughnia haani* (MW632159). Outgroups included two mysid species (*Neomysis japonica* (KR006340) and *N. orientalis* (KC995119), two euphausiid species (*Euphausia superba* (NC040987) and *E. pacifica* (NC016184)) and two amphipod species (*Metacrangonyx longipes* (HE861923) and *Caprella scaura* (NC014687)

The organization of the control region (CR) in stomatopods is known to consist of one or more GA-blocks, one or more poly-T stretches, several [TA(A)]n-blocks, and one or more hairpin structures which harbor the 5′ conserved motif “TTAT” [28]. The schematic depiction of all control regions belonging to twelve stomatopods (Fig. 4A), except that of *Oratosquilla oratoria* (superfam. Squilloidea), indicates that the CR is relatively well conserved among stomatopods. *Oratosquilla oratoria* does not possess a GA-3′-block, and [TA(A)]n-blocks are only located downstream (but not upstream) of the hairpin structure in this species [28].

**Fig. 5:**
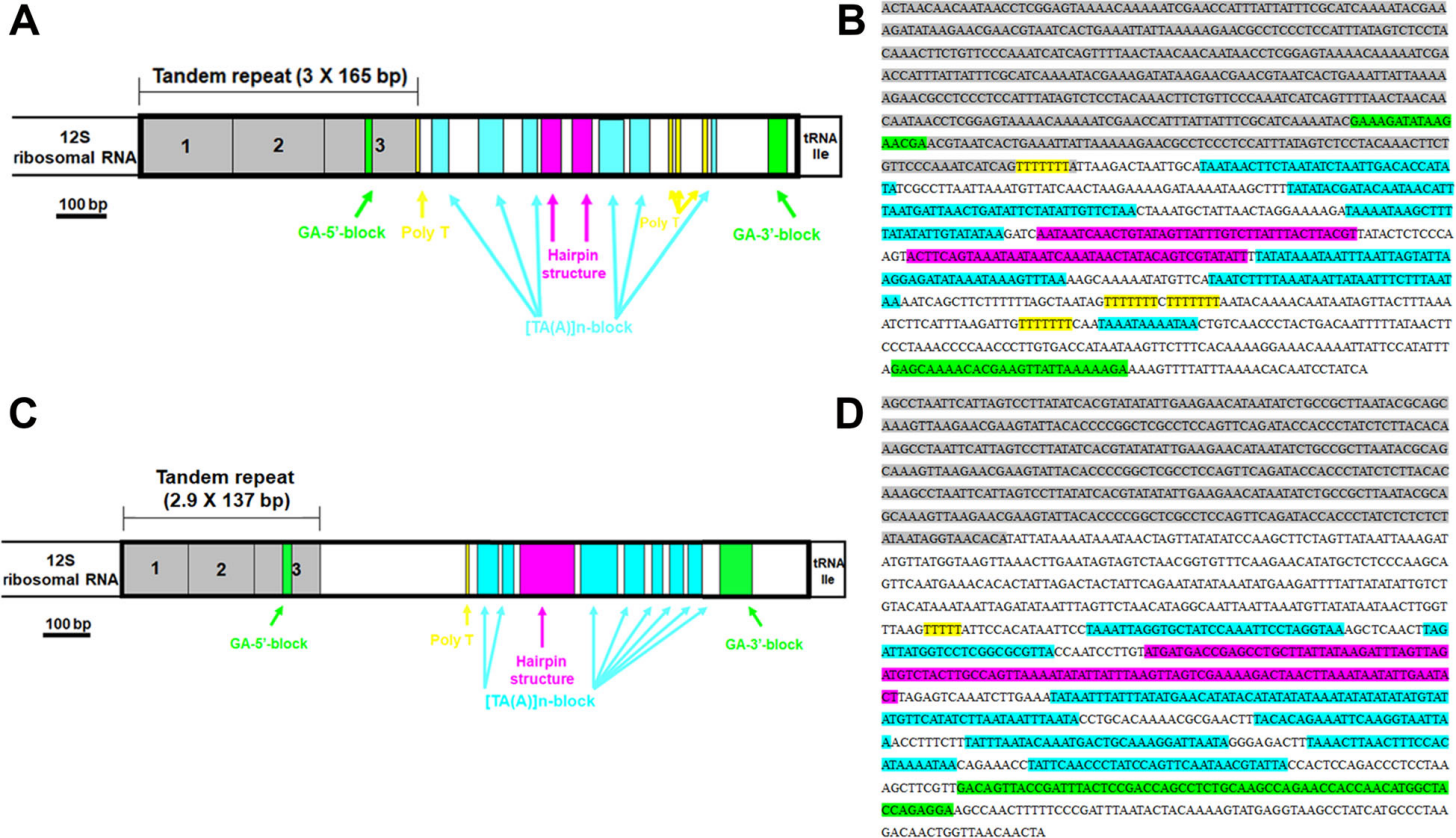
A, Structure of *Faughnia haani* control region. B, Sequence of *Faughnia haani* control region. C, Structure of *Lysiosquillina maculata* control region. D, Sequence of *Lysiosquillina maculata* control region. The putative conserved elements are highlighted: A-B, One fragment (165 bp long) found to repeat 3 times between 1 bp and 494 bp is shown in gray. Two GA-blocks (GA-5′-block and GA-3′-block) are marked in green. The poly-T stretch is marked in yellow, the hairpin structures are marked in pink. The [TA(A)]n-blocks which are known that can appear multiple times are marked with fluorescent blue. C-D, One fragment (137 bp long) found to repeat 2.9 times between 1 bp and 397 bp is shown in gray. Other elements are highlighted with the same color as mentioned above

A comparison of the D-Loop among superfamilies shows that parasquilloids are more similar to gonodactyloids than to squilloid and lysiosquilloid species because parasquilloids and gonodactyloids possess more poly-T stretches between the hairpin structures and [TA(A)]n-blocks than squilloids and lysiosquilloids (Fig. 4A). In detail, the control region of *F. haani* is relatively well organized, containing two GA-blocks (one GA-5′-block 17 bp in length starting at position 65 and another GA-3′-block 27 bp in length starting at position 1107); five 7-bp long poly-T stretches starting at positions 491, 920, 925, 933, and 985; and various [TA(A)]n-blocks starting at positions 514, 594, 672, 694, 867, and 995, flanking two different hairpins starting at positions 705 and 760 (Figs. 5A-B).

Visual examination of this CR, and the use of the web server Tandem Repeat Finder, enabled detection of one fragment which was 165 bp in length and was found to be repeated 3 times between positions 1 to 495 bp (Figs. 5A-B). A similar tandem repeat (3.1 × 137 bp) near the 5′ end of the control region has been reported before for the stomatopod *Lysiosquillina maculata* (superfam. Lysiosquilloidea) with structural similarity [24] (Figs. 5C-D). Additionally, a very similar tandem repeat has been found in other crustaceans (*Euphausia pacifica*: 4.7 × 154 bp [38] *Ligia oceanica*: 3.0 × 64 bp [27]) (Additional file 3) but not in all crustaceans (i.e., the Caribbean spiny lobster *Panulirus argus* [39]). Furthermore, the CR exhibited an imperfect inverted repeat located in positions 705–744 and 760–810 as indicated by EMBOSS: einverted. Multiple AT-rich dinucleotide and trinucleotide microsatellites along the entire stretch of the CR were detected using a microsatellite Repeat Finder (Additional file 2).

Finally, the web server RNAstructure predicted 20 secondary structures for the CR of *F. haani,* with ΔG values ranging from − 138.9 to − 137.8 kcal/mol. In all predicted secondary structures, several stem-loops were present along the entire length of the CR (Additional file 4).

### Mitophylogenomics of the Stomatopoda

The ML phylogenetic tree fully supports (bootstrap value = 100) the monophyly of the order Stomatopoda. Within the Stomatopoda, the analysis places the superfamily Parasquilloidea (represented by *F. haani*) in a sister position to all other representatives from the superfamilies Lysiosquilloidea, represented by *Lysiosquillina maculata* (fam. Lysiosquillidae), and Gonodactyloidea, represented by four species (*Taku spinosocarinatus*, *Gonodactylaceus randalli*, *Gonodactylus chiragra*, *Chorisquilla orientalis*) and three families (fam. Takuidae, Gonodactylidae, Protosquillidae), in our analysis. Furthermore, the tree places the superfamilies Parasquilloidea, Lysiosquilloidea, and Gonodactyloidea, in a sister position to the superfamily Squilloidea, represented by five genera and six species in the family Squillidae in our analysis (Fig. 4B).

Our results disagree with previous phylogenetic analyses that have indicated a monophyletic and sister relationship between the superfamilies Parasquilloidea and Squilloidea based on a larger number of species but a smaller number of molecular characters (i.e., fragments of the mitochondrial genes *cox1*, 12S, and 16S, and/or the nuclear 18S and 28S ribosomal RNA genes) [17, 18]. The phylogenetic position of the superfamily Parasquilloidea revealed by our study indicates that ‘spearing’ raptorial appendages are plesiomorphic, and that the ‘smashing’ type is either derived (in agreement with [18]) or apomorphic. Indeed, our phylogenetic analysis indicates that the spearing raptorial appendange may have independently evolved twice. Importantly, the superfamily Gonodactyloidea, characterized by the presence of a smashing raptorial appendage, has been recently confirmed to be polyphyletic [18, 19, 40, 41]. Thus, our results also suggest that the two types of raptorial appendages exhibited by stomatopods may represent evolutionary ‘labile’ characters, at least substantially more than originally thought [18, 19, 40, 41]. When considering vision, the Parasquilloidea is closely related to Gonodactyloids, with six midband rows of hexagonal ommatidia than Squilloidea which has the same number (two rows). Additional studies focusing on the assembly of mitochondrial genomes from parasquilloids as well as species belonging to different genera, families, and superfamilies within the order Stomatopoda are warranted before a more robust conclusion about the evolutionary history within this order can be deduced based on mitochondrial PCGs.

## Conclusions

This is the first genomic resource developed for a species belonging to the genus *Faughnia* and the superfamily Parasquilloidea and represents the first step to improving our understanding of the evolutionary history of stomatopods. Our results show that the superfamily Parasquilloidea is sister to the subfamilies Lysiosquilloidea and Gonodactyloidea. Additionally, the control region of the mitochondrial genome of *F. haani* provides useful phylogenetic information; it has structural similarities to that of the Gonodactyloidea. Our results are inconsistent with previous analyses that demonstrated a close relationship between Parasquilloidea and Squillioidea based on limited morphological and molecular traits. This study suggests that the spearing raptorial claw might have independently evolved twice. Our study provides insights into the phylogenetic relationships among superfamilies in the Stomatopoda and will guide future work to reveal the evolutionary history of visual elements and raptorial appendages in this iconic crustacean clade.

## Methods

### Field collection and sequencing

Genomic DNA (gDNA) was extracted from several walking legs of a single alcohol-preserved specimen collected in 2019 [42]. gDNA extraction was performed using the Qiagen DNeasy Tissue Kit (Qiagen, Hilden, Germany) following the manufacturer’s instructions. Next, mitochondrial DNA (mtDNA) amplification was performed using the gDNA as a template using the REPLI-g Mitochondrial DNA Kit (Qiagen, Hilden, Germany) according to the manufacturer’s instructions. The amplified mtDNA sample was quantified using the Quantus Fluorometer (Promega, USA) and was visualized using 1% agarose gel. After confirmation of mtDNA amplification, a DNA library was prepared using the NEXTflex Rapid DNA Sequencing Bundle Kit (PerkinElmer, USA). Sequencing was performed using the Illumina HiSeq 2500 platform with a 150-cycle protocol at the National Instrumentation Center for Environmental Management (NICEM), Seoul National University (SNU), Seoul, South Korea. A total of 3,724,799 reads were generated and rendered available in FASTQ format by the sequencing facility. All reads were used for the mitochondrial genome assembly of *F. haani.*

### Mitochondrial genome assembly of *Faughnia haani*

The mitochondrial genome of *F. haani* was assembled de novo using a two-step strategy. First, contigs were de novo assembled using the CLC assembler (Qiagen, Hilden, Germany). Next, we performed blast analysis of the newly assembled contigs against the mitochondrial genome of the closely related *Gonodactylaceus randalli* (MW019425). We selected the longest contig that was closest to the mitochondrial genome of *G. randalli* as a seed for de novo assembly with the pipeline NOVOPlasty version 3.8.2 [43] using a seed-and-extend algorithm to assemble organelle genomes from short-read raw datasets. During this final assembly, we used a word (= kmer) size of 22. NOVOPlastly successfully assembled and circularized the genome of *F. haani* with an average coverage of 508x per nucleotide.

### Mitochondrial genome annotation and analysis

The assembled mitochondrial genome was first annotated on the MITOS web server (http://mitos.bioinf.uni-leipzig.de) [44] using the invertebrate genetic code and again using Geneious Prime 2019.2.1 [45]. Annotation curation and start and stop codon corrections were performed using Expasy (https://web.expasy.org/) [46] and MEGA X [47]. Genome visualization was conducted with OrganellarGenomeDRAW (https://chlorobox.mpimp-golm.mpg.de/OGDraw.html) [48].

The nucleotide composition of the entire mitochondrial chromosome and of particular genes were estimated in MEGA X. The codon usage profile of each PCG was also analyzed. Codon usage for each PCG was predicted using the invertebrate code in the Codon Usage web server (http://www.bioinformatics.org/sms2/ codon_usage.html) [49]. tRNA genes were identified using the software MITFI [50] implemented in the web server MITOS. The secondary structure of each tRNA was predicted using the tRNAscan-SE v.2.0 web server (http://trna.ucsc.edu/tRNAscan-SE/) [50]. tRNA secondary structures were visualized using the Forna web server (http://rna.tbi.univie.ac.at/forna) [37].

The putative D-loop/CR of F. *haani* was examined in detail. The number of repeats in the region was investigated with the web server Tandem Repeat Finder v. 4.09 (http://tandem.bu.edu/trf/trf.html) [51]. DNA motifs were discovered in the putative D-loop/CR of F. *haani* using the default options in MEME [52]. The presence of inverted repeats was also detected using the web server ‘EMBOSS: einverted’ (http://www.bioinformatics.nl/cgi-bin/emboss/einverted) using the default options [53]. The existence and number of microsatellites (Simple Sequence Repeats, SSRs) were explored with the web server ‘Microsatellite repeats finder’ using the default options (http://insilico.ehu.es/mini_tools/microsatellites) [54]. Finally, the RNA structure web server (http://rna.urmc.rochester.edu/RNAstructureWeb/Servers/Predict1/Predict1.html) [55] was used to predict (using default options and a temperature of 27 °C) the secondary structure of this region, with special focus on the presence of stem-loops.

### Phylogenetic position of *Faughnia haani*

We examined the phylogenetic position of *F. haani* among other stomatopod species. The newly assembled and annotated mitochondrial genome of *F. haani* and those of a total of 12 stomatopod species available in the Genbank database were used for phylogenetic inference. Phylogenetic analyses included a total of 11 genera from 6 different families and 4 superfamilies in the order Stomatopoda. Outgroups included two mysid species (*Neomysis japonica* and *N. orientalis*), two euphausiid species (*Euphausia superba* and *E. pacifica*), and two amphipod species (*Metacrangonyx longipes* and *Caprella scaura*). For the analysis, the nucleotide sequences of each PCG were first translated into amino acids and aligned using Clustal W [56]. Next, poorly aligned regions were removed with trimAl [57]. The aligned sequences were concatenated, and the optimal dataset partition and best fitting models of sequence evolution were calculated using ModelFinder [58]. We used the ModelFinder results to conduct a maximum likelihood phylogenetic analysis in the software IQ-TREE [59]. The mtREV24 + G + I + F model was identified as the best-fit model, and the robustness of the ML tree topology was assessed by 1,000 bootstrap reiterations of the observed dataset.

## Supplementary Information

A**dditional file 1: Supplementary Table 1.** The respective overall base composition and length of stomatopod crustacean mitochondrial.

**Additional file 2. Supplementary Table 2.** Codon usage analysis of PCGs in the mitochondrial genome of Faughnia haani.

**Additional file 3. Supplementary Table 3.** Tandem repeats longer than 50 bp in the CR region of crustacean mitochondrial genomes.

**Additional file 4. Supplementary Figure 1.** Predicted secondary structure of the putative control region in Faughnia haani using the RNA structure web server.

## Data Availability

The newly obtained genome sequence data of *Faughnia haani* that support the findings of this study is available in GenBank (National Center for Biotechnology Information) at https://www.ncbi.nlm.nih.gov, accession no. MW632159. The associated BioProject, SRA, and BioSample numbers are PRJNA691084, SRR13414005, and SAMN17277782, respectively. The data are also available in Mendeley Data at 10.17632/mzk3mdnrwy.2. Also, the dataset analysed during the current study are available in the NCBI repository, accession no. NC007444 for *Squilla empusa*, accession no. NC006081 for *Squilla mantis*, accession no. NC027178 for *Squilloides leptosquilla*, accession no. MT276143 for *Lophosquilla costata*, accession no. NC014342 for *Oratosquilla oratoria*, accession no. NC006916 for *Harpiosquilla harpax*, accession no. MT672286 for *Chorisquilla orientalis*, accession no. MT672285 for *Taku spinosocarinatus*, accession no. DQ191683 for *Lysiosquillina maculata*, accession no. DQ191682 for *Gonodactylus chiragra*, accession no. MW019425 for *Gonodactylaceus randalli*, accession no. KR006340 for *Neomysis japonica*, accession no. KC995119 for *Neomysis orientalis*, accession no. NC040987 for *Euphausia superba*, accession no. NC016184 for *Euphausia pacifica*, accession no. HE861923 for *Metacrangonyx longipes*, and accession no. NC014687 for *Caprella scaura*.

## Abbreviations

Bp: Basepair
CR: Control region
PCG: Protein-coding gene
Leu: Leusine
Phe: Phenylalanine
Ile: Isoleucine
Pro: Proline
Cys: Cysteine
Arg: Arginine
Ser: Serine
fam.: Family
superfam.: Superfamily
