# Supplementary material for: The mitochondrial genome of Faughnia haani (Stomatopoda): novel organization of the control region and phylogenetic position of the superfamily Parasquilloidea
Source: BMC Genomics. 2021 Oct 2;22:716. doi: 10.1186/s12864-021-08034-x (PMC8487505; doi:10.1186/s12864-021-08034-x)
Supplement: Supplementary file 1 — Additional file 1: Supplementary Table 1. The respective overall base composition and length of stomatopod crustacean mitochondrial. [file 12864_2021_8034_MOESM1_ESM.pdf]

Supplementary information

• The Files are attached together below and therefore the form is .pdf

Additional file 1. The respective overall base composition and length of stomatopod crustacean mitochondrial genomes.

Additional file 2. Codon usage analysis of PCGs in the mitochondrial genome of *Faughnia haani*.

Additional file 3. Tandem repeats longer than 50 bp in the CR region of crustacean mitochondrial genomes.

Additional file 4. Predicted secondary structure of the putative control region in *Faughnia haani* using the RNA structure web server.

Additional file 1. The respective overall base composition and length of stomatopod crustacean mitochondrial genomes.

Species name	A + T	A	T	G	C	G+C	Length(bp)
Squilloides leptosquilla (NC02178)	71.20%	37.40%	33.80%	11.30%	17.50%	28.80%	15,995 bp
Oratosquilla oratoria (GQ292769)	70.80%	36.90%	33.90%	12.30%	16.90%	29.20%	15,783 bp
Squilla mantis (AY639936)	70.20%	35.10%	35.10%	13.00%	16.80%	29.80%	15.994 bp
Harpiosquilla harpax (AY699271)	69.80%	35.00%	34.80%	13.10%	17.10%	30.20%	15,714 bp
Chorisquilla orientalis (MT672286)	69.20%	35.50%	33.70%	12.40%	18.30%	30.70%	15,880 bp
Lophosquilla costata (MT276143)	68.00%	35.30%	32.70%	13.60%	18.30%	31.90%	15,771 bp
Gonodactylus chiragra (DQ191682)	67.50%	35.10%	32.40%	12.50%	20.10%	32.60%	16,279 bp
Faughnia haani (MW632159)	67.20%	35.30%	31.90%	13.30%	19.50%	32.80%	16,089 bp
Taku spinosocarinatus (MT672285)	66.80%	34.20%	32.60%	13.40%	19.80%	33.20%	15,960 bp
Gonodactylaceus_randalli (MW019425)	66.20%	33.20%	33.00%	14.70%	19.10%	33.80%	15,907 bp
Squilla empusa (DQ191684)	63.90%	32.80%	31.10%	14.70%	21.40%	36.10%	16,325 bp
Lysiosquillina maculata (DQ191683)	63.90%	32.80%	31.10%	14.70%	21.40%	36.10%	16,325 bp
