# Supplementary material for: The mitochondrial genome of Faughnia haani (Stomatopoda): novel organization of the control region and phylogenetic position of the superfamily Parasquilloidea
Source: BMC Genomics. 2021 Oct 2;22:716. doi: 10.1186/s12864-021-08034-x (PMC8487505; doi:10.1186/s12864-021-08034-x)
Supplement: Supplementary file 2 — Additional file 2. Supplementary Table 2. Codon usage analysis of PCGs in the mitochondrial genome of Faughnia haani. [file 12864_2021_8034_MOESM2_ESM.pdf]

Additional file 2. Codon usage analysis of PCGs in the mitochondrial genome of *Faughnia haani*.

AA	Codon	N	/1000	Freq	AA	Codon	N	/1000	Freq
Ala	GCG	25	6.92	0.11	Pro	CCG	9	2.49	0.07
	GCA	60	16.62	0.26		CCA	31	8.58	0.24
	GCT	94	26.03	0.41		CCT	55	15.23	0.42
	GCC	53	14.68	0.23		CCC	36	9.97	0.27
Cys	TGT	35	9.69	0.81	Gln	CAG	22	6.09	0.31
	TGC	8	2.22	0.19		CAA	49	13.57	0.69
Asp	GAT	41	11.35	0.54	Arg	CGG	10	2.77	0.18
	GAC	35	9.69	0.46		CGA	26	7.20	0.46
Glu	GAG	22	6.09	0.28		CGT	13	3.60	0.23
	GAA	57	15.79	0.72		CGC	8	2.22	0.14
Phe	TTT	221	61.20	0.74	Ser	AGG	28	7.75	0.09
	TTC	77	21.32	0.26		AGA	36	9.97	0.11
Gly	GGG	68	18.83	0.27		AGT	57	15.79	0.17
	GGA	75	20.77	0.30		AGC	11	3.05	0.03
	GGT	90	24.92	0.35		TCG	8	2.22	0.02
	GGC	21	5.82	0.08		TCA	43	11.91	0.13
His	CAT	40	11.08	0.48		TCT	106	29.35	0.33
	CAC	43	11.91	0.52		TCC	37	10.25	0.11
Ile	ATT	218	60.37	0.81	Thr	ACG	17	4.71	0.08
	ATC	51	14.12	0.19		ACA	69	19.11	0.33
Lys	AAG	24	6.65	0.30		ACT	73	20.22	0.35
	AAA	56	15.51	0.70		ACC	52	14.40	0.25
Leu	TTG	83	22.99	0.15	Val	GTG	34	9.42	0.14
	TTA	268	74.22	0.49		GTA	76	21.05	0.31
	CTG	26	7.20	0.05		GTT	109	30.19	0.44
	CTA	71	19.66	0.13		GTC	26	7.20	0.11
	CTT	75	20.77	0.14	Trp	TGG	24	6.65	0.25
	CTC	24	6.65	0.04		TGA	71	19.66	0.75
Met	ATG	43	11.91	0.20	Tyr	TAT	108	29.91	0.70
	ATA	176	48.74	0.80		TAC	47	13.02	0.30
Asn	AAT	71	19.66	0.56	End	TAG	1	0.28	0.08
	AAC	56	15.51	0.44		TAA	12	3.32	0.92
