# Supplementary material for: The mitochondrial genome of Faughnia haani (Stomatopoda): novel organization of the control region and phylogenetic position of the superfamily Parasquilloidea
Source: BMC Genomics. 2021 Oct 2;22:716. doi: 10.1186/s12864-021-08034-x (PMC8487505; doi:10.1186/s12864-021-08034-x)
Supplement: Supplementary file 3 — Additional file 3. Supplementary Table 3. Tandem repeats longer than 50 bp in the CR region of crustacean mitochondrial genomes. [file 12864_2021_8034_MOESM3_ESM.pdf]

Additional file 3. Tandem repeats longer than 50 bp in the CR region of crustacean mitochondrial genomes.

Class	Order	Species	GenBank accession No	Length (bp)	Copy no.	Reference
Malacostraca	Stomatopoda	*Faughnia haani*	MW632159	165	3	Present study
	Stomatopoda	*Lysiosquillina maculata*	NC007443	137	3.1	2
	Euphausiacea	*Euphausia pacifica*	EU587005	154	4.7	3
	Isopoda	*Ligia oceanica*	NC008412	64	3	4
Maxilopoda	Pentastomida	*Armillifer armillatus*	NC005934	387	4.9	5
	Cirripedia	*Pollicipes polymerus*	NC005936	118	2.8	5
Cephalocarida	Brachypoda	*Hutchinsoniella macracantha*	NC005937	135	3.3	5
			NC005937	121	2	5
