# Supplementary material for: The mitochondrial genome of Faughnia haani (Stomatopoda): novel organization of the control region and phylogenetic position of the superfamily Parasquilloidea
Source: BMC Genomics. 2021 Oct 2;22:716. doi: 10.1186/s12864-021-08034-x (PMC8487505; doi:10.1186/s12864-021-08034-x)

**Additional file 4.** Predicted secondary structure of the putative control region in *Faughnia haani* using the RNAstructure web server.

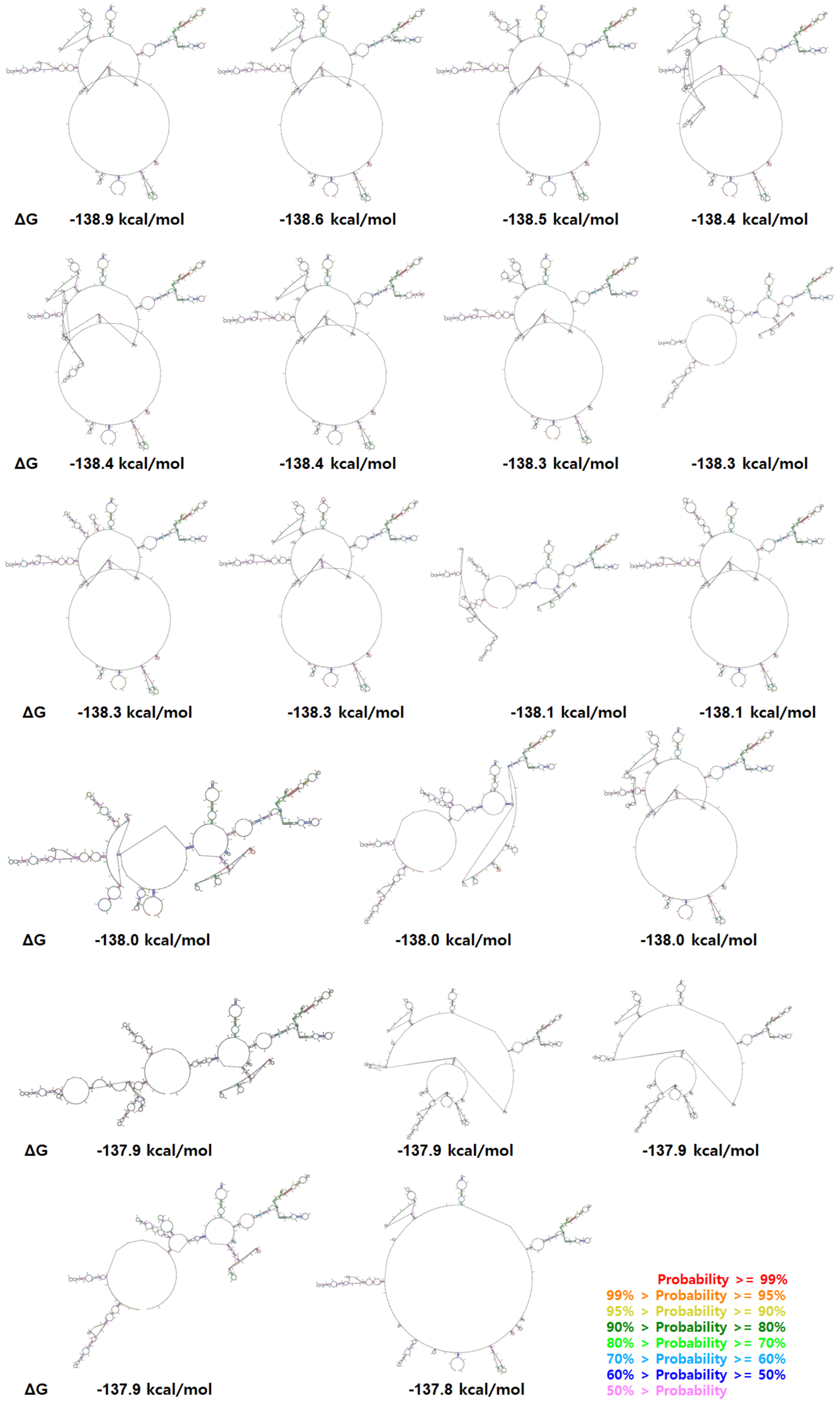

Supplement: Supplementary file 4 — Additional file 4. Supplementary Figure 1. Predicted secondary structure of the putative control region in Faughnia haani using the RNA structure web server. [file 12864_2021_8034_MOESM4_ESM.pdf]
